# Reduction of sodium content in spicy soups using monosodium glutamate

**DOI:** 10.3402/fnr.v60.30463

**Published:** 2016-06-27

**Authors:** Selamat Jinap, Parvaneh Hajeb, Roslina Karim, Sarian Norliana, Simayi Yibadatihan, Razak Abdul-Kadir

**Affiliations:** 1 Food Safety Research Centre (FOSREC), Faculty of Food Science and Technology, Universiti Putra Malaysia, Serdang, Malaysia; 2 Institute of Tropical Agriculture, Universiti Putra Malaysia, Serdang, Malaysia; 3 National Food Institute, Technical University of Denmark, Mørkhøj Bygade, Søborg, Denmark; 4 Department of Food Technology, Faculty of Food Science and Technology, Universiti Putra Malaysia, Serdang, Malaysia; 5 Department of Food Service, Faculty of Food Science and Technology, Universiti Putra Malaysia, Serdang, Malaysia

**Keywords:** MSG, NaCl, sodium reduction, spicy soup, saltiness, pleasantness

## Abstract

**Background:**

Excessive dietary sodium intake causes several diseases, such as hypertension, cardiovascular and renal disease, etc. Hence, reducing sodium intake has been highly recommended. In this study the effect of monosodium glutamate (MSG), as an umami substance, on saltiness and sodium reduction was investigated.

**Methods and Results:**

The trained panellists were presented with basic spicy soups (curry chicken and chili chicken) containing different amounts of sodium chloride (NaCl) (0–1.2%) and MSG (0–1.2%). They tasted the optimum concentrations of NaCl and MSG for the two spicy soups and the overall acceptability were 0.8% and 0.7%, respectively. There was no significant effect of spiciness level on the saltiness and umami taste of both soups. The optimum levels of combined NaCl and MSG for overall acceptance in the chili and curry soups were 0.3% and 0.7%, respectively. The results showed that with the addition of MSG, it is possible to reduce sodium intake without changing the overall acceptability of the spicy soup. A 32.5% reduction in sodium level is made feasible by adding 0.7% MSG to the spicy soups.

**Conclusions:**

This study suggests that low-sodium soups can be developed by the addition of appropriate amounts of MSG, while maintaining the acceptability of the spicy soups. It was also proven that it is feasible to reduce sodium intake by replacing NaCl with MSG.

## Practical Application

Saltiness and umami taste of soups are not affected by the level of spiciness. It is possible to reduce NaCl concentration and sodium intake in soup by replacing NaCl with MSG. The lower NaCl concentration of up to 62.5% and sodium intake of 32.5% was achieved by adding MSG to the spicy soups. Replacing NaCl with MSG in soups would contribute to better human health.

Sodium chloride (NaCl), which is added to foods as a preservative and a flavoring agent, is an important ingredient. Humans are genetically programmed to handle a NaCl intake of less than 0.25 g/day. Dietary Guidelines for Americans (DGA) recommends a sodium intake of less than 2,300 mg/day for normal people and around 1,500 mg for those aged 51 years and above, and individuals with hypertension, diabetes, or chronic kidney disease (1).

Recent dietary changes to a high NaCl intake present a major challenge to physiological systems to excrete these large amounts of NaCl through the kidneys (2). It has been found that excessive dietary intake of NaCl causes hypertension, cardiovascular disease, renal disease, etc. (3–5). Medical evidence indicates that reducing sodium intake lowers the blood pressure and decreases the incidence of diseases related to blood pressure (2, 6). Therefore, reducing sodium intake has been highly recommended, and various programs have been introduced in different countries to achieve gradual and sustained reductions in the amount of NaCl added to foods.

It is possible to replace some of the NaCl in foods with other taste or flavoring agents or through other flavor strategies or techniques. Different sodium substitutes, for example, potassium chloride (KCl), magnesium chloride (MgCl_2_), calcium chloride (CaCl_2_), monopotassium glutamate (MSG), calcium di-glutamate (CDG), potassium lactate, and glycine (7–10) have been used to reduce dietary sodium intake. Beyond sodium substitutes, there have been some naturally occurring or traditionally prepared foods (e.g. mushrooms, tomatoes, vegetable extracts) that might displace some of the need for added sodium in food preparation or manufacturing. However, reducing the NaCl content in foods may diminish their palatability. In this regard, it is important to find out an appropriate compound and its optimum levels which can reduce the NaCl content of foods without any negative effect on their palatability.

Glutamate, which is derived from glutamic acid, is an important contributor to food flavor. Studies have suggested that glutamate can be used for dietetic purposes to stimulate appropriate food choices in certain populations (11, 12). Institute of Medicine (US) Committee on Strategies to Reduce Sodium Intake (2010) has stated that a prominent example of an added compound involves the addition of the well-known flavoring compound MSG. This is because MSG imparts a savory taste (called ‘umami’) as well as a salty taste to foods (13). Usually, umami-rich stocks are recommended for preparing foods with reduced NaCl content (14, 15), as umami tastants in combination with NaCl can improve the acceptance of foods with reduced NaCl contents. Therefore, umami taste substances might be of value in maintaining the palatability of foods in which the NaCl content must be reduced.

MSG has been commercially manufactured since the early 1900s as a flavor enhancer to increase the sensory properties of foods, including palatability (16–18), richness, savoriness, and mouth-feel qualities (19–21). It is also generally recognized as a safe (GRAS) substance (22). It is possible to maintain food palatability with a lowered overall sodium level when MSG is substituted for some of the NaCl (23, 24). Hence, MSG has been suggested as a good flavor enhancer in low NaCl products that would not substantially increase the total sodium content of the product (14, 25). Accordingly, enhancing the overall flavor would increase the palatability and thus help surmount the period of initial loss of taste when NaCl is reduced.

The aim of the present study was to reduce the sodium content in spicy soups using MSG while maintaining their palatability. The sodium reduction was performed by investigating the effect of the level of spiciness on umami and saltiness of spicy soups, as well as the effect of MSG and NaCl levels on the umami taste of spicy soups and sodium reduction.

## Materials and Methods

### Materials

Standards of L-glutamate (minimum 99%-TLC, Sigma Aldrich, Steinhelm, Germany) and dansyl chloride (Assay ≥ 99.0%, Fluka, Buchs, Switzerland) were used. HPLC analytical grade solutions of glacial acetic acid and methanol were purchased from Merck (Darmstadt, Germany).

MSG was purchased from Ajinomoto (Malaysia) Berhad. Soup ingredients of common salt or NaCl, curry powder, chili powder, onion, garlic, ginger, coconut milk, lemon grass, lime, kaffir lime leaf, tamarind juice, mint leaf, chicken, fish, and cooking oil were purchased from hypermarkets in Selangor, Malaysia.

### Selection and training of the panelists

The untrained panelists consisted of 120 students and university staff. A total of 60 students and university staff who were non-smokers and had the experience of being a sensory panelist volunteered for the screening test for trained panelists. At the time of testing, none of the aspirants for trained panelists was on a severely restricted diet or suffered from any illnesses that could interfere with the perception of odors or tastes.

The sensory evaluation of the samples was carried out at the Sensory Laboratory of the Faculty of Food Science and Technology, Universiti Putra Malaysia. The trained panelists were selected through a series of basic taste tests, triangle tests, and paired-comparison tests. Sixteen panelists (2 males and 14 females) who answered more than 60% of the tests correctly and were able to perceive differences between the test samples were selected for further training. The training sessions were held before the actual sensory evaluation took place. The screened panelists went through training sessions that were conducted for 3 days per week for 2 months. The definition for the terminology of each taste was discussed and agreed upon by all the panelists during the training. They were trained with a series of standard solutions at different concentrations of NaCl (0–1.0%), MSG (0–1.0%), curry powder (0.5–2.5%), and chili powder (0.5–2.5%) dissolved in water. The concentration of salt and MSG in soup, curry powder, chili powder in water was based on weight/volume (w/v). They were then exposed to and familiarized with the curry and chili soups that were to be tested in the actual sensory analysis. To train the panelists for spiciness, soups containing 0.5% salt and 0.5% MSG with different levels of spices were used. They were trained to give the same score with a standard deviation of 10% for a given ingredient concentration.

### Preparation of soup stock and soup for sensory evaluation

Soup stock was prepared by boiling 200 g of chicken breast that had been cut into cubes (1 cm^3^) in 1 L of filtered water for 10 min. The soup was allowed to cool at room temperature and kept in the chiller (4°C) before being used. The soup stock was prepared one day before the sensory tests.

The soups for sensory evaluation were freshly prepared from the stock just before the sensory test sessions. About 50 mL of each sample was served in disposable polyethylene cups at 40°C, and the number of samples was not more than six per session. All samples were coded with a three-digit number and served randomly. Panelists were required to rinse out the mouth with warm water in between tasting.

### Determination of the optimum level of NaCl and MSG in spicy soups by untrained panelists

The soups with medium spiciness (containing 1% chili or curry) at different concentrations of NaCl (0.0–1.2%) and MSG (0.0–1.2%) were prepared. Medium level of spiciness was chosen because it was the level being recommended for consumers by the spice manufacturer. To determine the optimum level of NaCl and MSG, the untrained panelists were presented with both types of soups separately. They were asked to rate the overall acceptability of saltiness and umami taste for both the soups using a 9-point hedonic scale, with scores 1=‘dislike extremely’ and 9=‘like extremely’. The optimum levels of NaCl and MSG were chosen based on the highest score of acceptance. The optimum concentration was used to determine the effect of spiciness level on the intensity of saltiness and umami taste of both soups.

### Effect of NaCl–MSG combination on overall acceptability of spicy soups and sodium reduction

Untrained panelists were presented with both types of spicy soup mixtures at 40°C containing different percentages of NaCl (0–1%) and MSG (0–1%), at medium level of spiciness. Preliminary study on maximum concentration of pure solution of NaCl and MSG that can be tolerated by the trained panelists was found to be 1.0%. They were asked to evaluate the overall acceptability of the soups using the 9-point hedonic scale. Sodium reduction in spicy soups was calculated based on the following formula:

- Total weight of sodium in soup (g/100 mL), A: (x*22.99/58.44)+(y*22.99/169.11), where x=% NaCl added in the soup; y =% MSG added in the soup.
- Weight (g/100 mL) of sodium in optimum level of NaCl in soup (0.8%), B=0.8*22.99/58.44
- Reduction of sodium (%)=(A−B)/A*100

### Effect of spiciness on the intensity of saltiness and umami taste of spicy soups

Two different types of spicy soups were prepared by adding curry and chili powders to the soup stock. Different levels of the spices (0.5, 1, and 1.5% of soup stock) were stir-fried in 10 mL of vegetable oil for 2 min in a pot. Then, the soup stock was added to the spices followed by addition of optimum level of NaCl and MSG determined in the previous experiment. Then, the mixture was boiled for another 5 min before cooling at room temperature. The spiciness level was classified as low, medium, and high at 0.5, 1, and 1.5%, respectively. Only one type of spicy soup containing different concentrations of NaCl or MSG was presented to the panelists at one time. They were asked to rate the intensity of saltiness and umami taste on a line scale anchored at each end with 1=extremely weak and 9=extremely strong. Panelists were required to rinse out the mouth with warm water in between tasting.

### Statistical analysis

The results of the sensory attributes were statistically analyzed using analysis of variance (ANOVA) and the Duncan test to determine significant differences among the means at the level of p≤0.05 using the statistical package Minitab 14 statistical software (PA, State College, USA).

## Results

### Optimum levels of NaCl and MSG in spicy soups

**Fig. 1:**
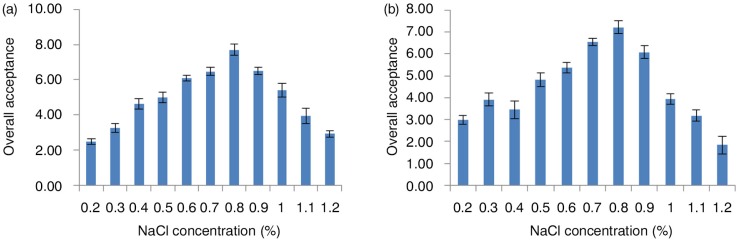
Overall acceptance of different concentrations of NaCl in curry chicken (a) and chili chicken (b) soups. NaCl: sodium chloride.

**Fig. 2:**
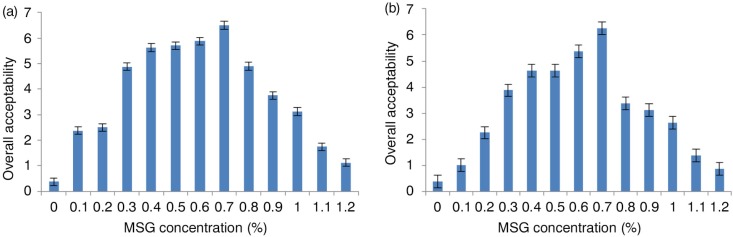
Overall acceptance of different concentrations of MSG in curry chicken (a) and chili chicken (b) soups. MSG: monosodium glutamate.

The effect of NaCl concentration on the overall acceptance of curry and chili soups is presented in Fig. 1a and b. Both curry and chili soups had an optimum level at NaCl concentration of 0.8%. The effect of MSG concentration on the overall acceptance of curry and chili soups is presented in Fig. 2a and b. The optimum MSG concentration was found to be 0.7% for both spicy soups. Generally, the overall acceptability increases as the concentration of NaCl or MSG increases up to optimum level, then it decreases significantly for each soup.

### Effect of NaCl–MSG combination on overall acceptability of spicy soups and sodium reduction

MSG was used to determine its effects on sodium reduction in spicy soups. To determine the optimum level, combinations of NaCl and MSG were applied to curry and chili soups and the panelists were asked to rate the soups for overall acceptance. Table 1 shows the scores given by the panelists to each soup prepared with different concentrations of NaCl and MSG. The ANOVA results (Table 1) indicated that the difference on the concentrations of NaCl and MSG in the NaCl–MSG combination yielded significant effects on the umami intensity of the soups. Generally, samples with higher concentrations of NaCl and MSG were highly preferred by the subjects. The overall acceptance ranged from 3.14 to 7.71 for the curry soup and from 3.2 to 7.71 for the chili soup (with a highest score of 9). The highest score for overall acceptance was given to the soups with 0.3% NaCl and 0.7% MSG. Overall, subjects were acceptive when the MSG concentration was increased, in spite of the NaCl concentration being decreased. These results indicated that by replacing 0.8% of the common NaCl in spicy soups with 0.3% NaCl and adding 0.7% MSG, the sodium content in curry and chili soups can be reduced by 32.5% (Table 1). These soups meet the FDA standards for a ‘reduced-sodium’ product (i.e. at least 25% less sodium than the original product) (7).

**Table 1:** ANOVA results for the overall acceptance of spicy soups with different concentrations of NaCl–MSG combination and their sodium reduction

	Intensity of umami (Mean±SD)		
		
NaCl/MSG concentration (%)	Curry chicken	Chili chicken	Sodium reduction (%)
0/1.0	3.14±0.86^h^	3.20±0.43^g^	57.1
0.1/0.9	4.14±0.77^fg^	4.14±0.57^f^	48.9
0.2/0.8	4.57±0.54^f^	4.64±0.95^e^	40.7
0.3/0.7	7.71±0.73^a^	7.71±0.63^a^	32.5
0.4/0.6	6.36±0.50^c^	6.36±0.50^c^	24.3
0.5/0.5	6.76±0.69^b^	6.93±0.63^b^	16.1
0.6/0.4	6.29±0.61^c^	6.36±0.63^c^	7.8
0.7/0.3	6.21±0.70^c^	6.23±0.60^c^	–
0.8/0.2	6.21±0.70^c^	6.29±0.53^c^	–
0.9/0.1	5.93±0.53^dc^	6.00±0.58^c^	–
1.0/0	5.29±0.53^e^	5.21±0.49^d^	–

Note: Score 1=dislike extremely; 9=like extremely.

MSG, monosodium glutamate; NaCl, sodium chloride; SD, standard deviation.

The superscript letters indicate the significance level of 0.05.

### Effect of spiciness on the saltiness and umami taste of spicy soups

Soups of different levels of spiciness (low, medium, and high) were prepared using the optimum concentration of NaCl and MSG observed earlier which were 0.8 and 0.7%, respectively. The percentage of NaCl/MSG mixture in soup was made up to 1.0% using NaCl/MSG based on the threshold concentration determined in the preliminary study. The panelists evaluated the intensity of saltiness and umami taste of both soups of different levels of spiciness. The results indicated that the different levels of spiciness did not show any significant differences (p<0.05) in terms of saltiness and umami for either soup (Table 2). The spiciness attributes of the studied soups ranged from 2.6 to 6.0 for the curry soup and from 4.2 to 6.3 for the chili soup.

**Table 2:** Taste intensity of umami and saltiness for curry chicken and chili chicken soups at different levels of spiciness

			Taste intensity (Mean±SD)	
			
NaCl/MSG concentration (%)	Type of soup	Spiciness level	Umami	Saltiness
0.8/0.2	Curry chicken	Low	5.87±0.12^a^	4.40±0.20^b^
		Medium	5.27±0.09^a^	4.94±0.13^b^
		High	5.20±0.17^a^	4.75±0.09^b^
	Chili chicken	Low	5.71±0.22^a^	4.69±0.09^b^
		Medium	5.40±0.18^a^	5.07±0.14^b^
		High	4.77±0.18^a^	4.56±0.12^b^
				
0.3/0.7	Curry chicken	Low	4.70±0.08^a^	3.82±0.09^b^
		Medium	5.09±0.11^a^	4.00±0.12^b^
		High	5.00±0.15^a^	4.36±0.12^b^
	Chili chicken	Low	5.73±0.23^a^	4.00±0.09^b^
		Medium	5.27±0.21^a^	4.82±0.12^b^
		High	5.00±0.16^a^	4.64±0.09^b^

Note: Letters a and b are the differences between rows for each taste.

MSG, monosodium glutamate; NaCl, sodium chloride; SD, standard deviation.

## Discussion

From the results, it can be seen that it is possible to reduce the NaCl concentration from 0.8 to 0.3% without changing the salty taste of soups by increasing the MSG concentration up to the optimum level of 0.7%. The non-significant effect (p>0.05) of different levels of spiciness on the saltiness and umami tastes of the soups prepared with 0.8/0.2% NaCl/MSG and 0.3/0.7% NaCl/MSG further proved that with the application of MSG, reduced concentrations of NaCl are possible without diminishing the overall acceptability of the soups (Table 2). A study reported by Ball et al. (9) also showed that the addition of MSG allows considerable reductions in the sodium content of simple pumpkin soup without a significant deterioration in taste. Other earlier studies that examined the interaction of salt and MSG in different types of soups also showed that it is possible to reduce sodium levels by substituting NaCl with MSG (17, 23, 26).

This study demonstrated that sodium concentrations can be reduced without negative effects on acceptance by the addition of MSG. According to these results, the salt concentration can be decreased to 0.3% without any significant change in the overall acceptance or saltiness of spicy soups by the addition of 0.7% MSG. This concentration is approximately 62.5% of the initial optimal concentration of NaCl (0.8%). Kremer et al. (27), who replaced salt with different concentrations of naturally brewed soy sauce, found NaCl reductions of 50, 17, and 29% in salad dressings, stir-fried pork, and soups, respectively. The foods they studied were still acceptable when the NaCl was substantially decreased and substituted with soy sauce. The study presented by Manabe (15) suggested that 17% of the NaCl in egg custard could be decreased by the addition of dried bonito fish. Mojet et al. (28) stated that MSG and inosine 5′-monophosphate can enhance the perceived saltiness of broth. Because MSG contains one third of the amount of sodium compared with that of table salt, it can be postulated that by replacing the same amount of NaCl with MSG, an approximately 40% reduction in the sodium content of soup can be achieved. Several studies have attempted to reduce the sodium levels in different types of foods, including soups (7), cheese (29), and meat products (30). However, the amount of sodium reduced has not been reported but rather the reduced percentage of NaCl has been discussed. Therefore, this study represents the only published comparison of the percentage of sodium reduction made possible by the addition of MSG. Compared with FDA standards for a ‘reduced-sodium’ product (i.e. at least 25% less sodium than the original product), the optimized soups are acceptable. In conclusion, the application of MSG as a NaCl substitute has successfully been shown to reduce sodium content.

It should be noted that in recent years, there have been some reports on possible adverse effects of MSG to some individuals who are sensitive to MSG. During early years, MSG was believed to be the cause of Chinese restaurant syndrome which is characterized by headache, flushing, numbness, muscle tightness, generalized weakness, and bronchoconstriction in asthmatics (31, 32). However, since the first report of this syndrome 50 years ago, clinical trials and recent studies have failed to identify a consistent relationship between the consumption of MSG and the constellation of symptoms that comprise the syndrome (33–35). Although there are reports that linked the consumption of glutamate to obesity, metabolic syndrome, and neurotoxic effects (36, 37), there have been no consistent data to support this relationship. The general consensus among scientists has been that glutamate is safe even in children, pregnant women, and lactating mothers (31, 38).

Besides, these adverse event reports triggered the official scientific organizations to examine the safety of MSG. Two major evaluations of the safety of MSG have been undertaken in recent history. The Joint FAO/WHO Expert Committee on Food Additives (JECFA) undertook an evaluation of MSG in 1987, and the Federation of American Societies for Experimental Biology (FASEB) undertook a review in 1995. The scientific reports identified some short-term, transient, and generally mild symptoms, as mentioned above may occur in some sensitive individuals who consume 3 g or more of MSG without food. In addition, they concluded there may be a small number of unstable asthmatics who respond to doses of 1.5–2.5 g of MSG in the absence of food (39, 40). In this regard, the dose of MSG (0.7%) used in this study is safe to be applied in the preparation of spicy soups, as the amount of MSG is considerably small, and it would be consumed in foods/soups.

## Conclusion

MSG and NaCl concentrations showed significant effects on the pleasantness, saltiness, familiarity, and taste intensity of spicy soups. The addition of MSG made it possible to reduce the NaCl concentration without affecting the pleasantness, saltiness, or taste intensity of the soups. The optimum overall acceptance of the NaCl/MSG combination in the spicy soups tasted by the trained panelists was achieved at 0.3 and 0.7% concentrations, respectively, which led to a 32.5% reduction in sodium intake. Soup is a common food that is majorly consumed all over the world. It is therefore essential to control the consumption level of NaCl in soups, in order to save consumers from the consequential health problems. Replacing NaCl with MSG in soup can contribute to better human health. Furthermore, MSG is an approved food enhancer and is listed in the GRAS list of food additives; such a substitution would not encounter significant regulatory obstacles, and the concentration used should be within the range of sensory preferences of the people who consume the foods.
